# Common biomarkers of Alzheimer disease and postoperative delirium

**DOI:** 10.1097/MD.0000000000048921

**Published:** 2026-05-29

**Authors:** Yiqi Fang, Xiao Zhang, Gaofeng Zhan

**Affiliations:** a Department of Anesthesiology, Chun’an First People’s Hospital, Zhejiang Provincial People’s Hospital Chun’an Branch, Hangzhou Medical College Affiliated Chun’an Hospital, Hangzhou City, Zhejiang Province, China; b Department of Anesthesiology, Chun’an Second People’s Hospital, Zhejiang Provincial People’s Hospital Chun’an Branch, Hangzhou Medical College Affiliated Chun’an Hospital, Hangzhou City, Zhejiang Province, China.

**Keywords:** Alzheimer disease, biomarker, decision tree, machine learning, postoperative delirium

## Abstract

Alzheimer disease (AD) and Postoperative delirium (POD) may share a common mechanism, but their shared genes and potential novel therapeutic targets remains unknown. We selected datasets GSE48350, GSE4226, and GSE16759 containing AD and control samples from the GEO database for AD model development, and use the GSE63061 dataset for AD model validation. The GSE163943 dataset containing PODs and controls was used to identify common genes. Differentially expressed genes (DEGs) associated with POD and AD respectively, as well as common DEGs between 2 diseases were analyzed. Shared DEGs were assessed using gene ontology and KEGG enrichment and PPI interaction networks. Machine learning algorithms, nomogram and decision tree analyses were used to interpret top 5 genes and visualize the models. 259 DEGs in POD with 148 being up-regulated and 111 down-regulated, and 199 DEGs in AD with 25 up-regulated and 174 down-regulated were found, of which 23 were common to POD and AD. Gene ontology analysis indicated that DEGs were primarily concentrated in 9 distinct biological processes, while KEGG results revealed 3 major metabolic pathways. Five hub genes emerged as potential diagnostic biomarkers for both diseases, Bruton tyrosine kinase (BTK), NCF2, CRH, FCGR3A, and SERPINA3. The LASSO model determined that the 23 common genes could serve as biomarkers for both POD and AD patients, and the Random Forest model is the the most suitable model. POD or AD decision trees showed BTK and NCF2 were sufficient to distinguish between POD or AD patients and healthy controls. BTK and NCF2 were identified common biomarkers of POD and AD. Further investigations are required to verify correlations between gene expression levels and pathological characteristics.

## 1. Introduction

Alzheimer disease (AD) is a neurodegenerative disease estimated to affect 26.8% of the population.^[1]^ Patients with AD exhibit clinical manifestations such as memory and cognitive impairments, aphasia, and personality changes, adversely affecting both patients and their families.^[2–4]^ AD-affected brains are characterized by extracellular plaques composed of β-amyloid (Aβ), and intracellular filamentous tangles resulting from Tau hyperphosphorylation, leading to synaptic damage and neurological dysfunction.^[5]^

Postoperative delirium (POD) is an acute and transient organic mental disturbance syndrome which is characterized by sudden onset, cognitive impairment, decreased attention span, increased or decreased mental activity, and disrupted sleep patterns.^[6]^ Studies have reported that the incidence of POD is between 9% and 41%.^[7,8]^ Recent research has investigated the presence of AD biomarkers in POD, and studies have confirmed the expression of Aβ, Tau-PT217, and Tau-PT181 in POD patients.^[9–11]^ However, it is still unclear whether the pathogenesis of POD is linked to molecular changes in AD, as well as the correlation and potential mechanisms between the 2. In addition, since the incidence of POD in AD patients is higher,^[12]^ identifying common genes between the 2 can help in the search for preventive and treatment drugs for POD in AD patients.

This study used bioinformatics methods to screen for common key genes in the pathogenesis of AD and POD, and searched for key biomarkers and potential drugs, offering insights for clinical practice.

## 2. Methods

### 2.1. Data extraction and preprocessing

This study was approved by the Ethics Committee of Chun’an Second People’s Hospital, Zhejiang Provincial People’s Hospital Chun’an Branch. Three GEO datasets were used for AD model development. Of these GSE48350, included 80 AD samples with 173 healthy controls, GSE4226 comprised 80 AD and 14 control samples, and GSE16759 included 4 AD and 4 controls. A further dataset, GSE63061, comprising 139 AD and 134 control samples, was used for model validation, while the GSE163943 dataset, including 4 POD and 4 control samples, was utilized to screen for common hub genes. The expression value or PM value of a probe set in a sample was divided by the median of the expression values or PMs of that probe set across all samples, then logged to reflect the consistency of parallel experiments. For the raw datasets GSE48350, GSE4226, and GSE16759, preprocessing was conducted with the RAM method. Regression analyses were conducted on 5 raw datasets utilizing the R packages affyPLM and RColorBrewer.

### 2.2. Differentially expressed genes, GO and KEGG analyses, and PPI networks

Genes fulfilling the criteria of logFC]1 and FDR[0.05 were considered differentially expressed genes (DEGs) (where FC represents fold change and FDR is the false discovery rate). The top 10 up-regulated and down-regulated genes were determined. Clustering analysis to visualize the DEGs was performed using volcano plots and heatmaps. The Venn diagram illustrated the common DEGs across the datasets. Data were analyzed using R version 3.6.1.

The R WGCNA package was utilized for construction of a gene co-expression network, and genes from network modules most strongly associated with clinical features were utilized for gene ontology (GO) and KEGG analyses, using a Benjamini correction at *P* < .05. These genes represented candidate biomarkers.

A protein–protein (PPI) network of DEGs was established using STRING (v11.0) with a combined confidence score threshold of 0.4. Hub genes were identified. The network was visualized using Cytoscape.

### 2.3. Machine learning algorithms, nomogram and decision tree analyses

gene set enrichment analysis was used to perform 1000 permutation analyses on the expression matrices to obtain the enrichment results. LASSO regression was employed to identify the most predictive genes and construct a diagnostic signature based on feature selection.

index = i = 1n genei * coefi

We validated the LASSO model through the POD dataset GSE163943 and the AD dataset GSE63061. Model performance was evaluated using ROC curves, with the area under the curve (AUC) value representing the primary performance metric. Random Forest (RF) and Support Vector Machine (SVM) models were established using LASSO-selected features and compared via ROC analysis, residual distribution assessment, and boxplot visualization. We selected the top 5 genes to form a signature according to their ranking importance, and validated it using the same datasets. A nomogram for the top 5 genes was constructed, with higher scores indicating a higher probability of POD and AD. Calibration curves and decision tree models were used for further interpretation and visualization of the models.

Given the relatively limited sample size of the POD dataset, the machine learning models developed in this study should be considered exploratory analyses aimed at identifying potential shared biomarkers between AD and POD. These models provide preliminary insights into candidate diagnostic genes, but their predictive performance requires further validation in larger independent datasets.

## 3. Results

### 3.1. Data preprocessing

The median value of RLE for each sample is around 0, and the median value of NUSE is around 1, indicating no outlier samples and the dataset was of good quality, which could be included in the analysis.

### 3.2. Screening of DEGs

**Figure 1.**
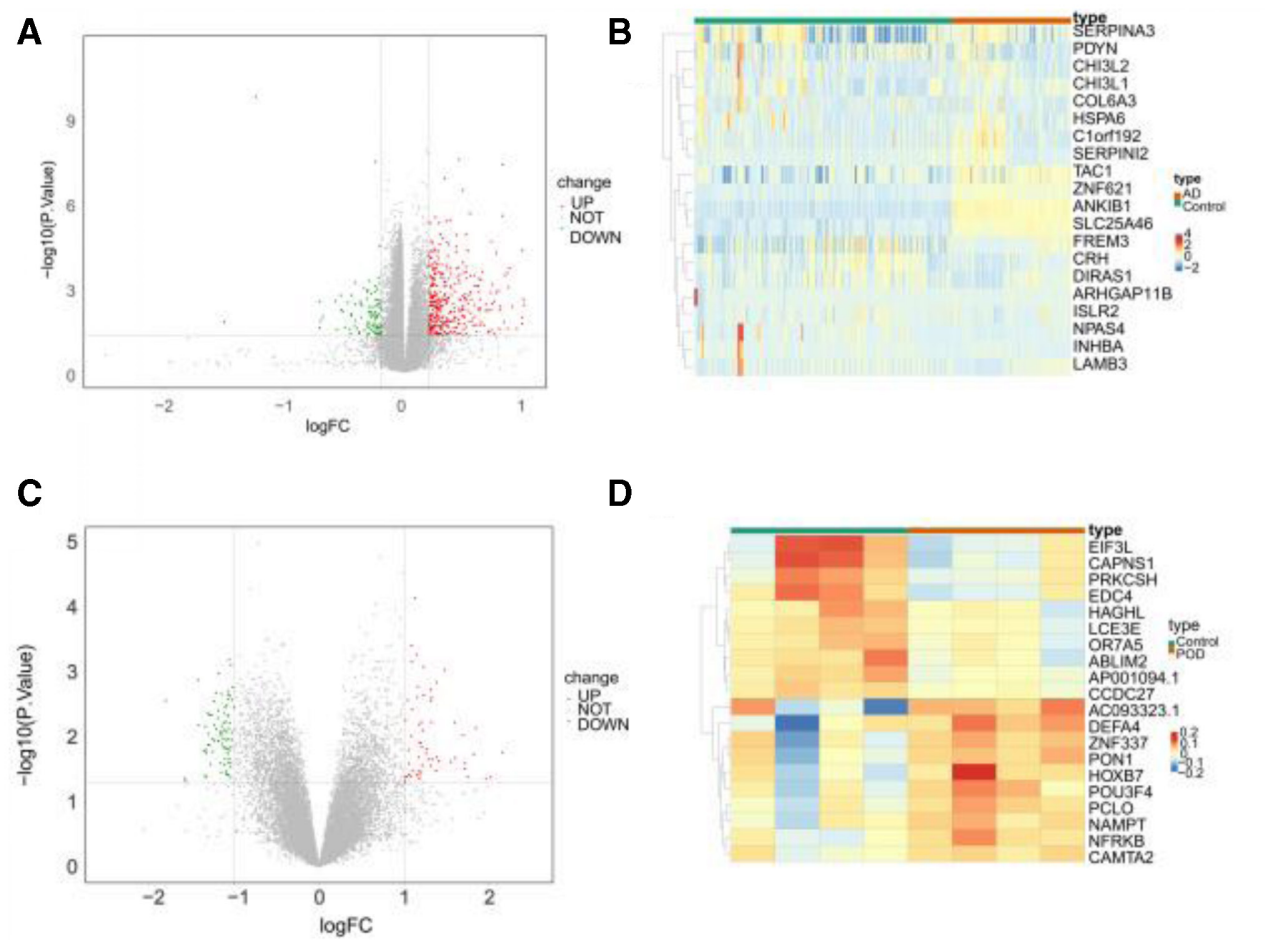
Identification of DEGs in Alzheimer disease and postoperative delirium. (A) Volcano plot of DEGs in AD patients. (B) Hierarchical clustering heatmap of DEGs in AD patients. (C) Volcano plot of DEGs in POD patients. (D) Hierarchical clustering heatmap of DEGs in POD patients. AD = Alzheimer disease, DEGs = differentially expressed genes, POD = postoperative delirium.

Overall, 22,880 genes were identified in the AD and normal groups. These included 199 DEGs, of which 25 were up-regulated and 174 down-regulated (Fig.1A). The top 10 up- and down-regulated DEGs are illustrated in the heatmap (Fig. 1B). Similarly, 41,115 genes were found in the POD and normal groups, including 259 DEGs, with 148 showing up-regulation and 111 down-regulation (Fig.1C). The heatmap of the top 10 up- and down-regulated DEGs is shown in Figure 1D.

**Figure 2.**
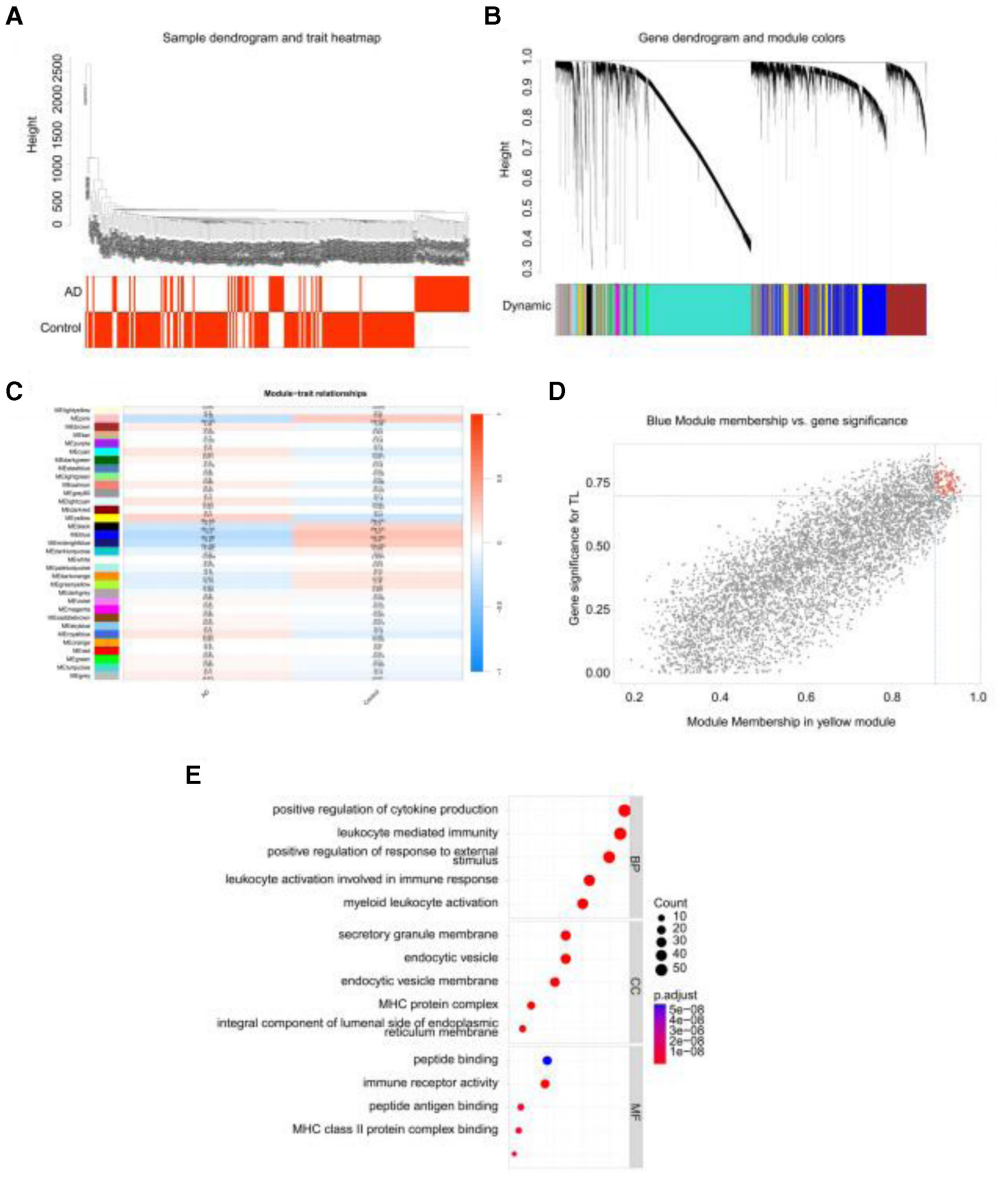
WGCNA identifies core genes associated with AD clinical traits. (A) Sample dendrogram and trait heatmap. (B) Gene dendrogram and module colors. (C) Module-trait association heatmap. (D) Scatter plot of gene significance versus module membership for identifying hub genes. (E) GO functional enrichment analysis of the co-expression module. AD = Alzheimer disease, DEGs = differentially expressed genes, GO = gene ontology, POD = postoperative delirium, WGCNA = weighted gene co-expression network analysis.

After integrating the AD data, gene variances were determined and the top 75% genes were analyzed further. The hclust function removed the deviant samples (GSM300301) (Fig. 2A). Twenty gene modules were identified with different colors, among which the gray module represented genes that were not classified into any module (Fig. 2B). After extracting the characteristic genes of each module, the heatmap showed that the blue module was most strongly associated with AD clinical outcomes (Fig. 2C). Genes with high correlation (gene significance 0.7) and module membership 0.9) were selected, yielding 69 candidate genes including 19 DEGs (Fig. 2D). Functional annotation revealed enrichment in immune response pathways (cytokine signaling, leukocyte activation), cytoskeletal components, and antigen processing, implicating immune dysregulation as a central mechanism in AD pathogenesis (Fig. 2E).

**Figure 3.**
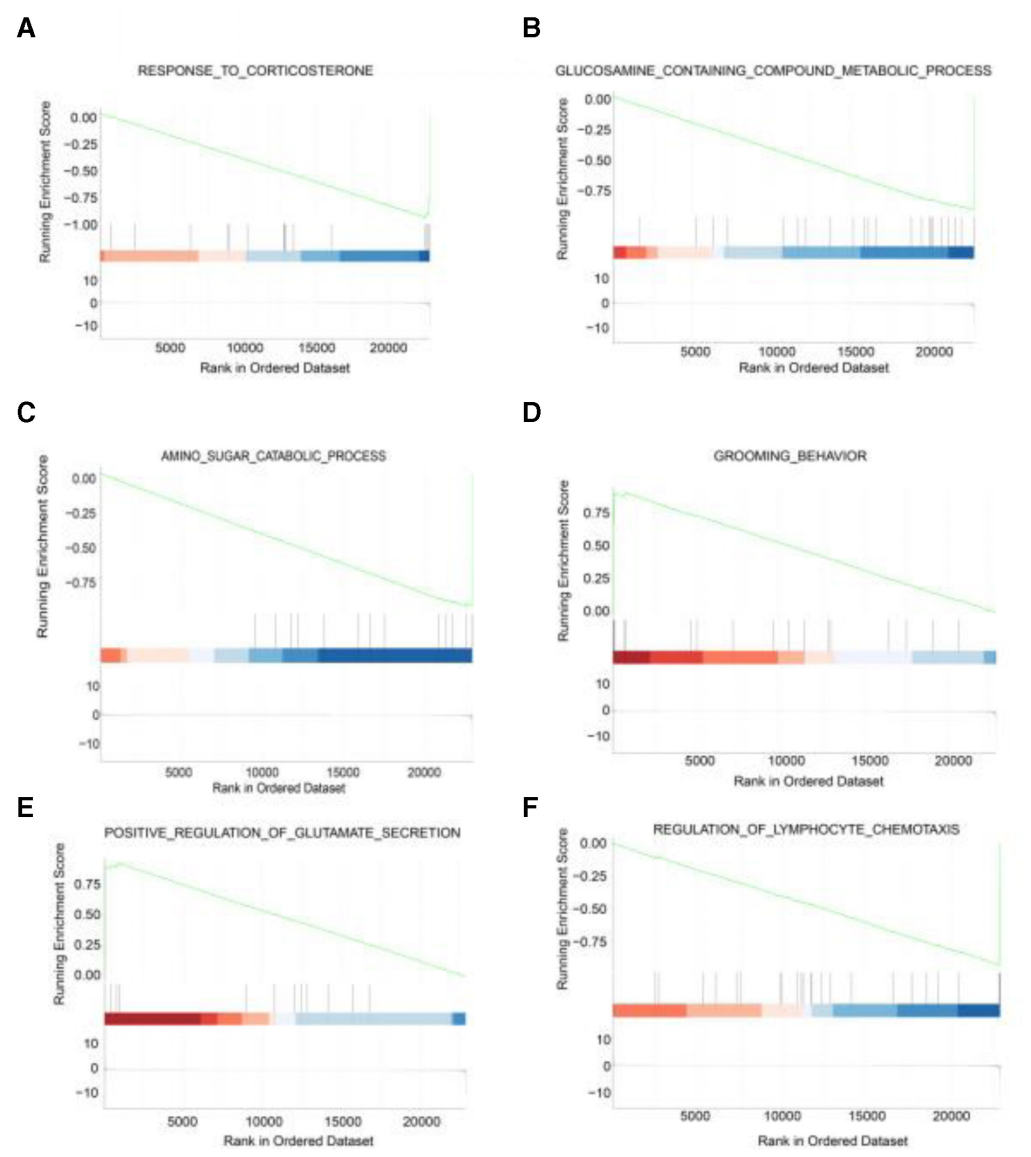
GSEA reveals down-regulated pathways in AD patients. (A) GSEA plot for “Response to corticosterone” pathway. (B) GSEA plot for “Glucosamine containing compound metabolic process” pathway. (C) GSEA plot for “Amino sugar catabolic process” pathway. (D) GSEA enrichment plot for the “grooming behavior” gene set. (E) GSEA enrichment plot for the “positive regulation of glutamate secretion” gene set. (F) GSEA plot for “Regulation of lymphocyte chemotaxis” pathway. AD = Alzheimer disease, GSEA = gene set enrichment analysis

Gene set enrichment analysis identified dysregulated metabolic and behavioral pathways in AD, with down-regulated pathways predominantly related to carbohydrate metabolism, immune regulation, and neurotransmitter signaling (Fig. 3).

### 3.3. Enrichment analysis of DEGs common to POD and AD

**Figure 4.**
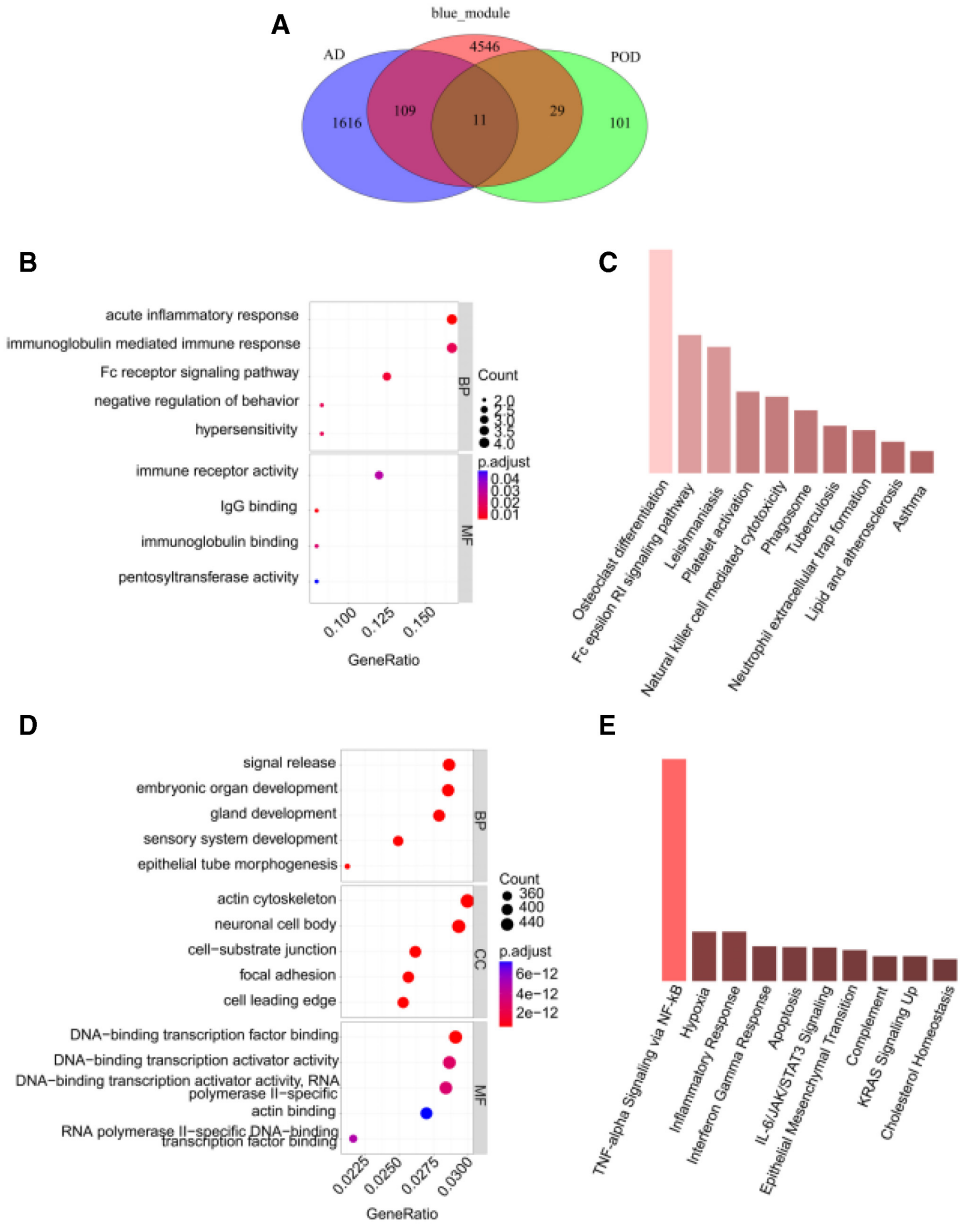
Enrichment analysis reveals common dysregulated genes and pathways in POD and AD. (A) Venn diagram showing overlapping genes among co-expression module, POD DEGs, and AD DEGs. (B) GO enrichment analysis of genes common to all 3 sets. (C) KEGG pathway enrichment analysis of genes common to all 3 sets. (D) GO enrichment analysis of AD-specific differential genes. (E) KEGG pathway enrichment analysis of AD-specific differential genes. AD = Alzheimer disease, DEGs = differentially expressed genes, GO = gene ontology, KEGG = kyoto encyclopedia of genes and genomes, POD = postoperative delirium.

The Venn diagram was drawn for the genes of AD blue module, POD DEGs, and AD DEGs, showing an overlap of 11 genes (Fig.4A). Comprehensive functional annotation of common DEGs using GO and KEGG revealed convergent enrichment in immune-related biological processes and pro-inflammatory pathways. The prevalent functional themes included acute inflammatory responses, immunoglobulin-mediated immunity, and Fc receptor signaling at the biological process level, B-cell and immune receptor-associated activities at the molecular function level, and immune cell compartments and cytoskeletal structures at the cellular component level (Figs. 4B-D). KEGG pathway analysis further identified enrichment in critical inflammatory cascades (TNF-α/NF-κB, hypoxia response) and immune differentiation pathways (osteoclast differentiation), collectively implicating dysregulated immune responses and neuroinflammation as central pathogenic mechanisms in both AD and POD (Fig.4E).

### 3.4. LASSO model construction

**Figure 5.**
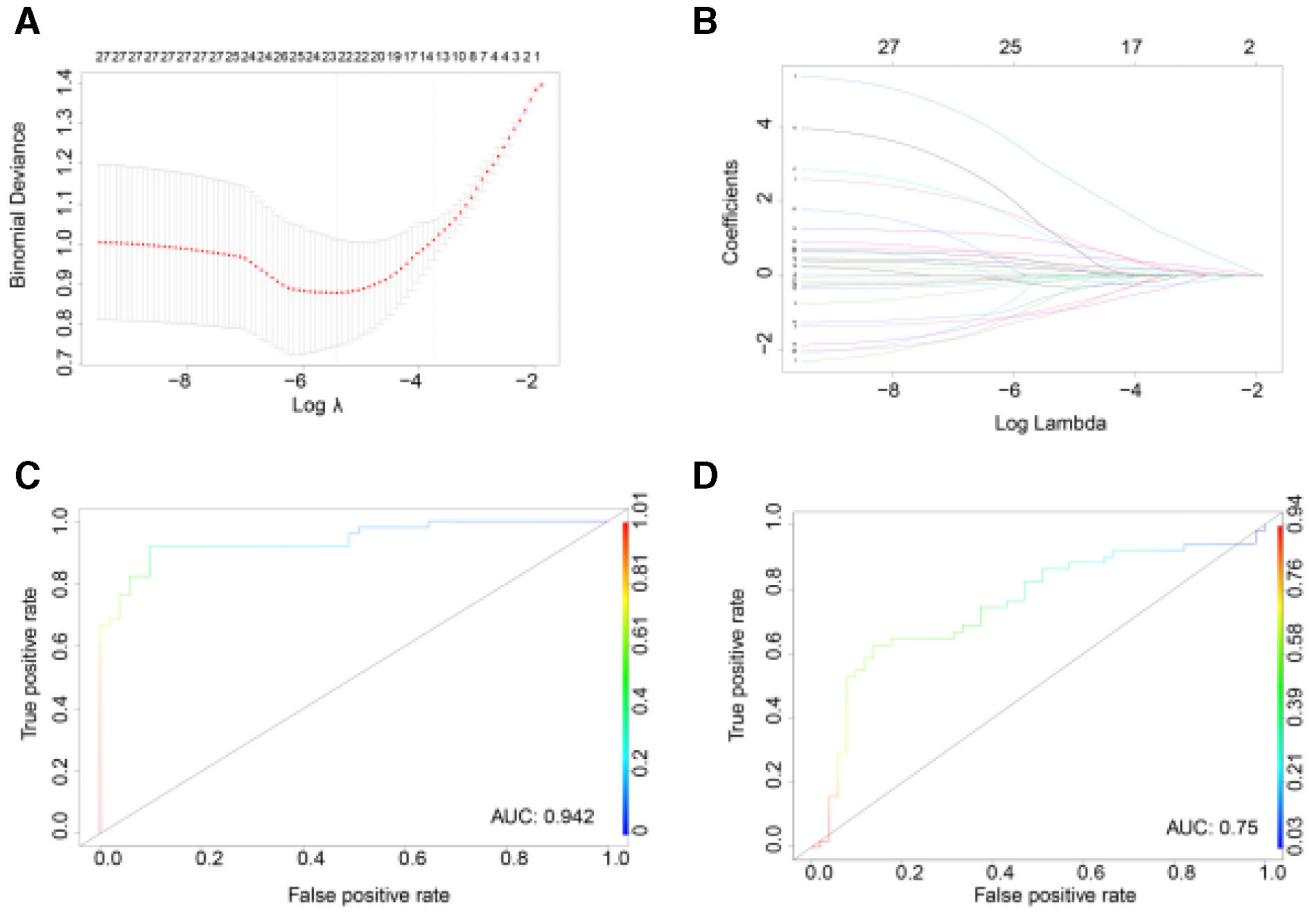
Lasso regression model construction and validation for identifying common biomarkers of POD and AD. (A) Lasso coefficient path plot showing regularization of gene coefficients. (B) Cross-validation error plot for Lasso model optimization. (C) ROC curve of Lasso model in POD dataset (AUC = 0.942). (D) ROC curve of Lasso model in AD dataset (AUC = 0.75). AD = Alzheimer disease, AUC = area under the curve, POD = postoperative delirium, ROC = receiver operating characteristic.

The WGCNA analysis identified 19 genes showing significant associations with AD. Using LASSO, the optimal λ value was selected to create a signature that included 23 genes (Figs. 5A-B). The ROC curve showed an AUC of 0.942 in the POD dataset (Fig. 5C) and an AUC of 0.75 in the AD dataset (Fig. 5D), indicating that the model based on these 23 genes can be used to identify both POD and AD patients.

### 3.5. SVM and RF models

**Figure 6.**
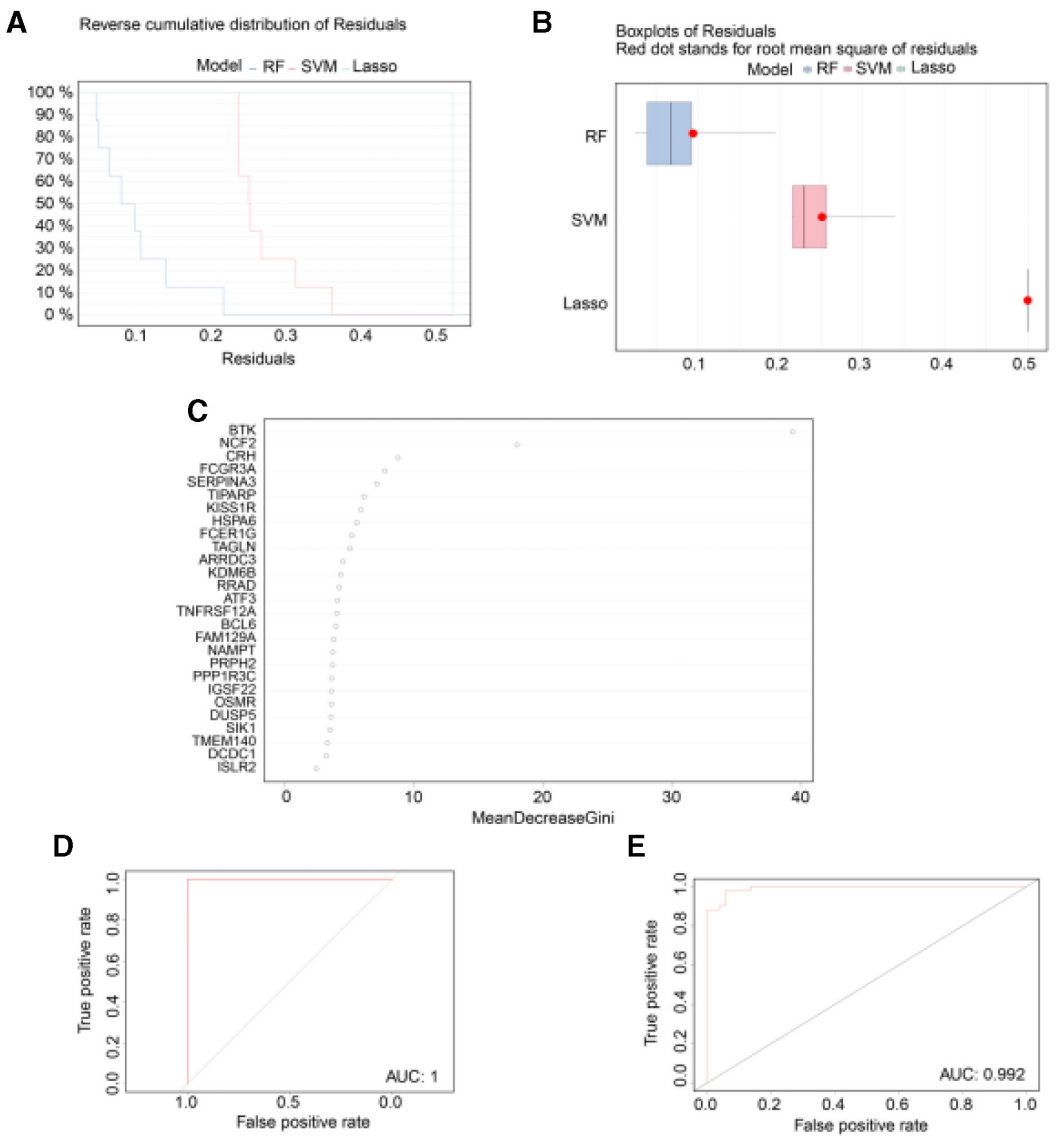
Comparison of machine learning models and identification of optimal biomarker signature. (A) Reverse cumulative distribution of residuals comparing 3 models (RF, SVM, Lasso). (B) Boxplots of residuals showing root mean square errors for each model. (C) Feature importance ranking of top genes in the Random Forest model. (D) ROC curve of Random Forest model in POD dataset (AUC = 1.0). (E) ROC curve of Random Forest model in AD dataset (AUC = 0.992). AD = Alzheimer disease, AUC = area under the curve, POD = postoperative delirium, RF = random forest, ROC = receiver operating characteristic, SVM = support vector machine.

The RF model showed smaller residuals in comparison with the SVM and LASSO models (Figs. 6A-B). Consequently, the RF model was deemed the most suitable. The top 10 most significant genes are illustrated in Figure 6C. The RF model was validated in both the POD and AD datasets, with ROC curves showing AUC values of 1 for POD and 0.992 for AD (Fig. 6D).

### 3.6. Nomogram construction and decision tree analysis

**Figure 7.**
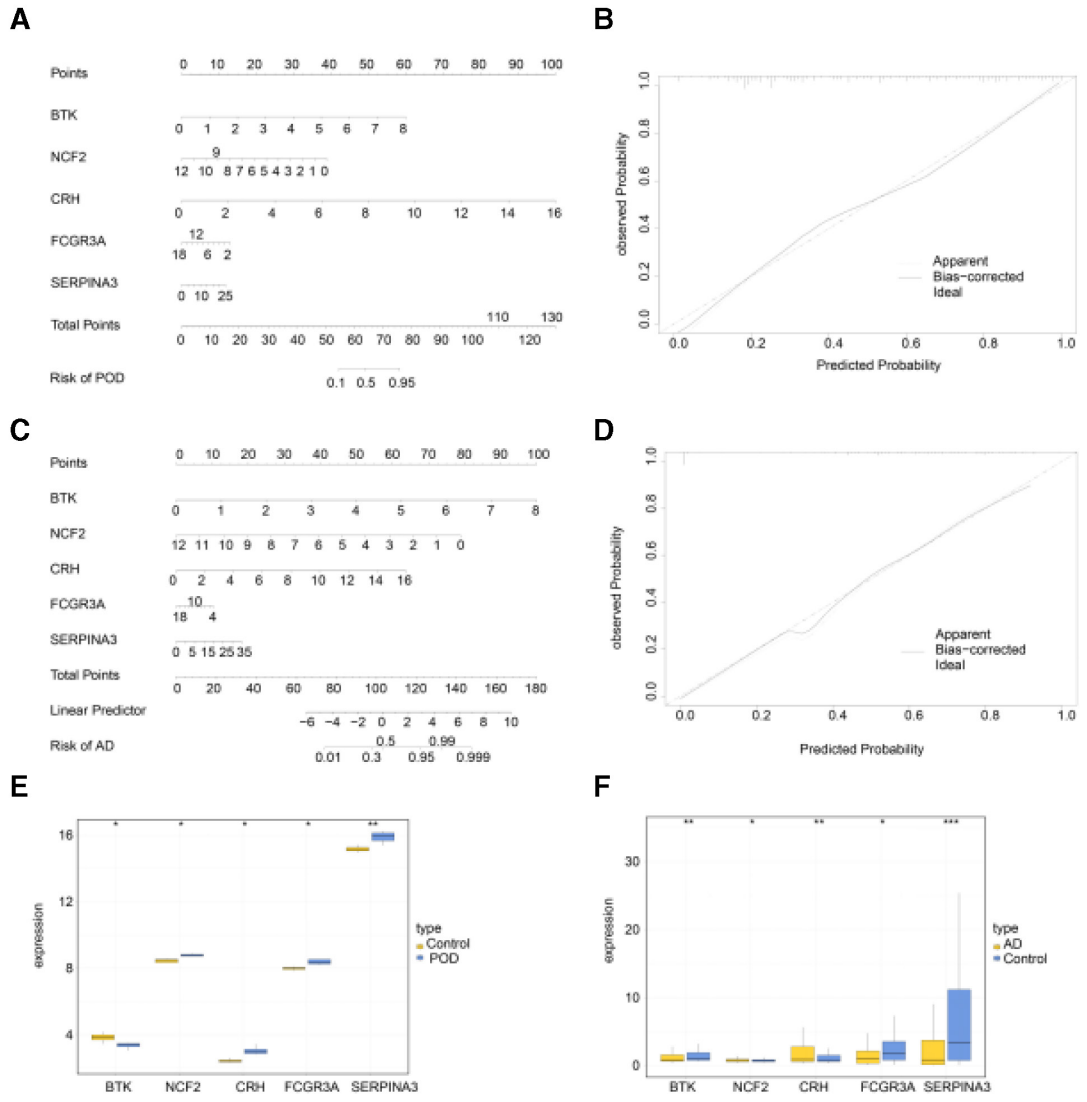
Nomogram construction and decision tree analysis for POD and AD risk prediction. (A) Nomogram for POD risk prediction based on 5 hub genes (BTK, NCF2, CRH, FCGR3A, SERPINA3). (B) Calibration curve for POD nomogram. (C) Nomogram for AD risk prediction based on 5 hub genes. (D) Calibration curve for AD nomogram. (E) Expression levels of 5 hub genes in POD patients versus healthy controls. (F) Expression levels of 5 hub genes in AD patients versus healthy controls. AD = Alzheimer disease, BTK = bruton tyrosine kinase, CRH = corticotropin-releasing hormone, FCGR3A = Fc gamma receptor IIIa, NCF2 = neutrophil cytosolic factor 2, POD = postoperative delirium, SERPINA3 = serpin family A member 3.

The top 5 genes, BTK, CRH, NCF2, FCGR3A, and SERPINA3, were selected for the construction of individual nomograms (Fig 7A, C). These nomogram models could accurately predict the positivity rates for POD and AD (Fig 7B, D). In POD patients, BTK was down-regulated, while NCF2, CRH, FCGR3A, and SERPINA3 were up-regulated (Fig. 7E). In AD patients, BTK, FCGR3A, and SERPINA3 were down-regulated, while NCF2 and CRH gene expressions were up-regulated. In the POD or AD decision tree, only BTK and NCF2 expression was needed to distinguish POD/AD patients from healthy controls (Fig. 7).

## 4. Discussion

In this study, four AD datasets and 1 POD dataset were selected, and through bioinformatics analysis, DEGs associated with the diseases, as well as shared DEGs, were identified. Five genes that were most closely connected in the PPI network and located in important node positions were extracted, namely, BTK, NCF2, CRH, FCGR3A, and SERPINA3. The SVM model of these genes showed high accuracy in diagnosing AD and POD, while decision tree analysis indicated the core contributions of BTK and NCF2.

Bruton tyrosine kinase (BTK) is a non-receptor tyrosine kinase associated with the B-cell receptor and mediating B-cell proliferation and differentiation.^[13,14]^ It is a central component of pathways involving cell surface receptors, including the B-cell antigen receptor, which is activated in secondary lymphoid organs, driving proliferation of cancerous B cells, such as in chronic lymphocytic leukemia. BTK inhibitors are currently tending to replace chemotherapy in treating chronic lymphocytic leukemia and similar diseases, and are also utilized in treating Waldenström ’ s macroglobulinemia, small lymphocytic and marginal zone lymphomas, and chronic graft-versus-host disease.^[15]^ According to Mansour HM’s research, COVID-19 and AD share pathogenic cofactors, making patients with AD more vulnerable to the disease with more severe symptoms and consequences. Tyrosine kinases such as BTK are associated with interactions between the immune and nervous systems.^[16]^

The NCF2 gene encodes neutrophil cytoplasmic factor 2, a 67-kDa component of the NADPH oxidase complex in neutrophils. The complex is responsible for superoxide production, associated with neutrophil phagosomes. Mutations in NCF2 and other components of the oxidase complex are linked to chronic granulomatous disease, associated with recurrent infections. The gene is alternatively spliced, producing multiple isoforms. Studies by Li et al suggest that the involvement of ALOX5 and NCF2 in the formation of necrotic cores in AS by modulating ferroptosis in macrophages.^[17]^ Research by Li et al indicates that changes in NCF2 and NOX2 mRNA levels in patients with PTB, suggesting the involvement and diagnostic potential of these genes in PTB.^[18]^ As reported by Wang et al, NCF2 is associated with apoptosis in AD.^[19]^ Greve et al showed that, at the mechanistic level, DEP administration elevated Tnf (Tnf α), Ncf1 (p47PHOX), and Ncf2 (p67PHOX) mRNA expression in Trem2^+/+^mice, while (IL-1β) levels were only raised in Trem^2-/-^ mice. This emphasizes the crucial role of Trem2 in neuroinflammation in DEP-treated mice, suggesting the involvement of TREM2 in the effects of air pollution neuroinflammation, with implications for associations between air pollution and AD.^[20]^

From a mechanistic perspective, BTK and NCF2 may participate in a shared pathological axis linking perioperative stress, neuroinflammation, and cognitive impairment. Surgical trauma and anesthesia are known to trigger systemic inflammatory responses and activate innate immune pathways. BTK signaling has been implicated in microglial activation and inflammatory signal amplification in the central nervous system. Meanwhile, NCF2, as a component of the NADPH oxidase complex, contributes to the production of reactive oxygen species, which are closely associated with oxidative stress and neuronal injury. The coordinated activation of BTK-mediated immune signaling and NCF2-dependent oxidative pathways may synergistically enhance neuroinflammatory responses, ultimately promoting synaptic dysfunction and cognitive decline observed in both AD and POD.

Enrichment analysis findings substantiate the role of immune dysregulation and metabolic dysfunction in AD pathogenesis. GO profiling identified significant enrichment in fundamental biological processes (signal transduction, developmental pathways) and immune cell-related components (cytoskeletal machinery, cell-cell contact sites), with emphasis on transcriptional regulatory activities governing immune responses. Pathway analysis via KEGG highlighted 2 complementary mechanisms: (1) pro-inflammatory signaling cascades (TNF-α/NF-κB pathway, hypoxia-responsive genes), and (2) metabolic reprogramming in immune cells(osteoclast differentiation and related pathways).

Through data mining and bioinformatics analysis of POD and AD datasets, this study had identified 2 hub genes, BTK and NCF2, which were closely linked to both POD and AD. Additionally, it had been found that physiological processes andpathways such as extracellular matrix remodeling and 3/2 signaling, are associated with both POD and AD, providing a foundation for further research into POD and AD mechanisms and therapeutic targets. However, further investigation is required to verify the specific functions of these genes identified here in the development of these diseases.

Despite the promising findings, several limitations should be acknowledged. First, the sample size of the POD dataset (GSE163943) is relatively small (4 POD patients and 4 controls), which may limit the statistical power and generalizability of the machine learning models. Small sample sizes may increase the risk of overfitting and reduce the robustness of biomarker identification. Therefore, the predictive performance of the models reported in this study should be interpreted with caution. Future studies using larger multicenter datasets and independent clinical cohorts are necessary to validate the diagnostic value and biological relevance of the identified biomarkers.

In conclusion, BTK and NCF2 were identified common biomarkers of POD and AD offering insights for clinical practice. Nonetheless, further investigation is needed to confirm the relationships between gene expression and clinical pathological characteristics.

## Acknowledgments

We thank all the researchers in department of Anesthesiology, Chun ‘an first People’s Hospital.

## Author contributions

**Conceptualization:** Yiqi Fang, Xiao Zhang, Gaofeng Zhan.

**Data curation:** Yiqi Fang, Xiao Zhang, Gaofeng Zhan.

**Formal analysis:** Yiqi Fang, Xiao Zhang, Gaofeng Zhan.

**Funding acquisition:** Gaofeng Zhan.

**Investigation:** Gaofeng Zhan.

**Writing – original draft:** Yiqi Fang, Gaofeng Zhan.

**Writing – review & editing:** Yiqi Fang, Gaofeng Zhan.
